# Supplementary material for: Exosomes Derived from Hypoxia-Cultured Human Adipose Stem Cells Alleviate Articular Chondrocyte Inflammaging and Post-Traumatic Osteoarthritis Progression
Source: Int J Mol Sci. 2023 Aug 29;24(17):13414. doi: 10.3390/ijms241713414 (PMC10487932; doi:10.3390/ijms241713414)
Supplement: Supplementary file 1 [file ijms-24-13414-s001.zip › ijms-2563593-supplementary.pdf]

Supplementary Figure S1

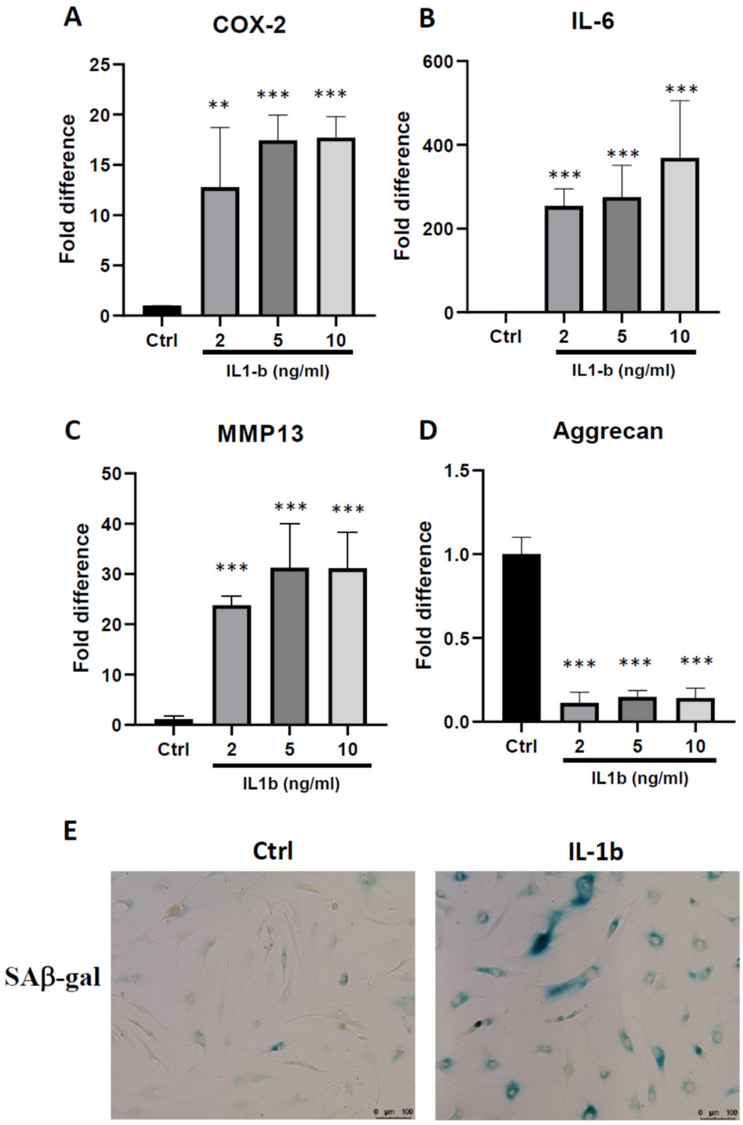

Figure S1. IL-1β induced OA-like inflammaging in articular chondrocytes.

A. COX-2, B, IL-6, C, MMP13, and D, Aggrecan mRNA expression in HACs treated with IL-1β (2, 5, 10 ng/mL) for 2 days in vitro. GAPDH mRNA expression was used as the internal control. Each column represents the mean ± SEM of three replicate cultures. Data from each group were compared with those of the control group, **, $P < 0.01$; ***, $P<0.001$ compared to the control culture. E. Senescence-associated β-galactosidase (SA-β-gal) staining to verify IL-1β (10 ng/mL)-induced OA-like articular chondrocyte senescence. The left panel shows the control culture, and the right panel shows the articular cartilage treated with IL-1β for 12 days (scale bars: 100 μm).

Supplementary Table S1

Table S1. Primer sequences used for real-time polymerase chain reaction.

Gene	Forward (5 ′ → 3′)	Reverse (5 ′ → 3′)
COL2a1	CAA CAC TGC CAA CGT CCA GAT	TCT TGC AGT GGT AGG TGA TGT TCT
Aggrecan	ACA GCT GGG GAC ATT AGT GG	GTG GAA TGC AGA GGT GGT TT
COL10a1	AGC CAG GGT TGC CAG GAC CA	TTT TCC CAC TCC AGGAGGGC
PTHrP	ATG CAG CGG AGA CTG GTT CAG CAG T	GGG AGA GGG CTT GGA GTT AGG GG
ADAMT5	GCT ACT GCA CAG GGA AGA GG	TGC ATA TTT GGG AAC CCA TT
MMP13	TCT TGA GCT GGA CTC ATT GT	AGC CTC TCA GTC ATG GAG CT
CEBPB	GAC AAG CAC AGC GAC GAG TA	AGC TGC TCC ACC TTC TTC TG
PTGS2	TGA GCA TCT ACG GTT TGC TG	TGC TTG TCT GGA ACA ACT GC
IL1-b	ATG ATG GCT TAT TAC AGT GGC AA	GTC GGA GAT TCG TAG CTG GA
IL6	AAG ATG GCT GAA AAA GAT GGA TGC	GTT GGG TCA GGG GTG GTT ATT G
TNFa	CCC AGG GAC CTC TCT CTA ATC	ATG GGC TAC AGG CTT GTC ACT
GAPDH	TCT CCT CTG ACT TCA ACA GCG AC	CCC TGT TGC TGT AGC CAA ATT C

Cycling conditions: Denature: 95°C for 30 s, 95°C for 4 min, followed by 35 cycles of 95°C for 10 s, 55–61°C (shown in column of Annealing Temperature) for 15 s and 72°C for 15 s
